# Real-world practice patterns and outcomes for RAI-refractory differentiated thyroid cancer

**DOI:** 10.1530/ETJ-23-0039

**Published:** 2024-01-24

**Authors:** Andrew G Gianoukakis, Jennifer H Choe, Daniel W Bowles, Marcia S Brose, Lori J Wirth, Taofeek Owonikoko, Svetlana Babajanyan, Francis P Worden

**Affiliations:** 1 The Lundquist Institute at Harbor-UCLA Medical Center, David Geffen School of Medicine at UCLA, Torrance, California, USA; 2 Department of Medicine, Duke University Medical Center/Duke Cancer Institute, Durham, North Carolina, USA; 3 Division of Medical Oncology, University of Colorado, Aurora, Colorado, USA; 4 Department of Otorhinolaryngology: Head and Neck Surgery, Abramson Cancer Center of the University of Pennsylvania, Philadelphia, Pennsylvania, USA; 5 Harvard Medical School, Massachusetts General Hospital, Boston, Massachusetts, USA; 6 Winship Cancer Institute of Emory University, Atlanta, Georgia, USA; 7 Bayer HealthCare Pharmaceuticals, Whippany, New Jersey, USA; 8 Comprehensive Cancer Center, University of Michigan, Ann Arbor, Michigan, USA

**Keywords:** advanced differentiated thyroid cancer, active surveillance, real-world evidence, ATA risk stratification

## Abstract

**Background:**

The optimal timing for initiating multi-kinase inhibitors (MKIs) in patients with radioactive iodine-refractory (RAI-R) differentiated thyroid cancer (DTC) remains unclear. Thus, we evaluated the real-world practice patterns and outcomes in asymptomatic patients with progressive RAI-R DTC (≥1 lesion ≥1 cm in diameter) in the USA (US population) and outside the USA (non-US population).

**Methods:**

In this prospective, non-interventional, open-label study, eligible patients were chosen by treating physicians to receive MKI therapy (cohort 1) or undergo active surveillance (cohort 2) at study entry. Cohort 2 patients were allowed to transition to MKI therapy later. The primary endpoint was time to symptomatic progression (TTSP) from study entry. Data were compared descriptively. When endpoints were inestimable, 36-month rates were calculated.

**Results:**

Of the 647 patients, 478 underwent active surveillance (cohort 2) and 169 received MKI treatment (cohort 1). Patients underwent surveillance at a higher rate in the US (92.6%) vs the non-US (66.9%) populations. Half of US and non-US patients who qualified for MKI treatment had initial American Thyroid Association (ATA) low-to-intermediate-risk disease. In cohort 2, the 36-month TTSP rates from study entry were 65.6% and 66.5% in the US and non-US populations, respectively. Cohort 2 patients treated later demonstrated 36-month TTSP rates of 30.8% and 55.8% in the US and non-US populations, respectively.

**Conclusions:**

Active surveillance is a viable option for asymptomatic patients with progressive RAI-R DTC. However, early intervention with MKI therapy may be more suitable for others. Further research is needed to identify patients who are optimal for active surveillance.

**Registration:**

NCT02303444.

## Introduction

Differentiated thyroid cancer (DTC) accounts for nearly 90% of all thyroid cancer cases (1). The standard initial treatment is surgery followed by radioactive iodine (RAI) or observation; however, approximately 5–15% of patients become refractory to RAI therapy (2). The prognosis for these patients is poor, and the 10-year survival rate for RAI-refractory (RAI-R) thyroid cancer is 10% (3).

Multi-kinase inhibitors (MKIs) are the standard of care for RAI-R DTC. While MKIs are not curative, they can improve progression-free survival (PFS) (4, 5) and prolong the duration of response (6). Careful consideration must be given to selecting patients for MKI therapy, including the timing of treatment initiation. MKIs can significantly lower a patient’s quality of life (4, 5, 7, 8), and RAI-R patients can be asymptomatic, progression-free, or have slowly progressive disease for several years (8). Therefore, patients with stable or indolent disease should be considered for active surveillance. Real-world data suggest that delaying MKI therapy until rapid disease progression may result in a suboptimal clinical benefit (9). However, there is no consensus on when to initiate MKI therapy.

To elucidate the optimal timing for administering MKIs, we aimed to evaluate the real-world practice patterns and long-term outcomes of asymptomatic patients with progressive RAI-R DTC in the USA (US population). Our study investigated two groups of patients: those under active surveillance and those who received multi-kinase inhibitor (MKI) therapy at the time of study entry based on the treating physician’s discretion. Furthermore, a qualitative comparison was made between the findings in the US group and those from outside of the USA (non-US population).

## Materials and methods

### Study design

The RIFTOS MKI study (NCT02303444) was a non-interventional, prospective, open-label study conducted in 19 countries across 92 sites. Data collection occurred from April 2015 to July 2020. US data was kept separate from non-US data to be analyzed separately. At study entry, eligible patients chosen for MKI therapy by their treating physician were enrolled in cohort 1, while patients chosen for initial surveillance by their treating physician were enrolled in cohort 2. Cohort 2 patients were allowed to transition to MKI therapy later.

The study adhered to the guidelines and regulations of the European Medicines Authority, the US Food and Drug Administration, and local laws and regulations. In the USA, the protocol was reviewed and approved by the Western Institutional Review Board (WIRB). Participating sites were required to subject their informed consent forms for site specific approval. When required, 77 sites used their local IRB. All patients provided written informed consent before enrollment.

### Patient selection

Eligible patients had asymptomatic RAI-R DTC and showed radiological progression, with at least one lesion ≥1 cm. RAI-R status was defined based on previously reported criteria (10). Patients who were previously treated with MKIs, enrolled in clinical trials, or in hospice care were excluded. A minimum life expectancy of six months was required. Survival information was collected even if patients joined an interventional clinical trial during the observation period.

### Endpoints and assessments

The primary endpoint was the time to symptomatic progression (TTSP) from study entry. Symptomatic progression encompassed metastasis-related bone symptoms, respiratory symptoms, neurologic events, bleeding, discomfort or pain, declining general health, or reduced mobility or death from any cause. Secondary endpoints were PFS and overall survival (OS) from study entry of the overall population (cohorts 1 and 2 combined) and cohort 2 alone. TTSP, PFS, and OS were also assessed according to cohort 2 subgroups: age, American Thyroid Association (ATA) risk stratification after surgery (herein referenced as initial ATA risk), median sum of the longest diameter of target lesions prior RAI dose, and MKI treatment post enrollment.

TTSP was met when patients developed symptoms attributed to the site of existing metastatic lesions. This required confirmation by radiological examination of the DTC lesion at the symptomatic location. Patients who remained asymptomatic until analysis were censored at their last evaluable assessment date. Those who joined an interventional trial during the study were censored at their last evaluable assessment prior to trial enrollment. Patients without any follow-up after study entry were censored on day 1.

### Statistical analysis

Patient demographic, clinical, and treatment characteristics were analyzed descriptively, using counts, percentages for categorical variables, medians, and interquartile ranges (IQRs) for continuous variables. Bivariate analyses using propensity score matching were planned to balance the study cohorts; however, due to insufficient patient enrollment, the planned propensity score matching was not conducted. Consequently, no statistical comparisons between cohorts were performed. Instead, data were analyzed descriptively using Kaplan–Meier methods to estimate median PFS, OS, and TTSP along with their corresponding 95% CIs. Since the median was not reached for many parameters, the 36-month PFS, OS, and TTSP rates were calculated.

Additionally, we conducted a *post hoc* subgroup analysis, stratifying patients from cohort 2 based on age, initial ATA risk, sum of the longest target lesion(s) diameter, prior RAI dose, and MKI treatment status post enrollment.

Data were not analyzed for US cohort 1 alone because only 13 patients were enrolled.

## Results

### Patient sample

**Figure 1:**
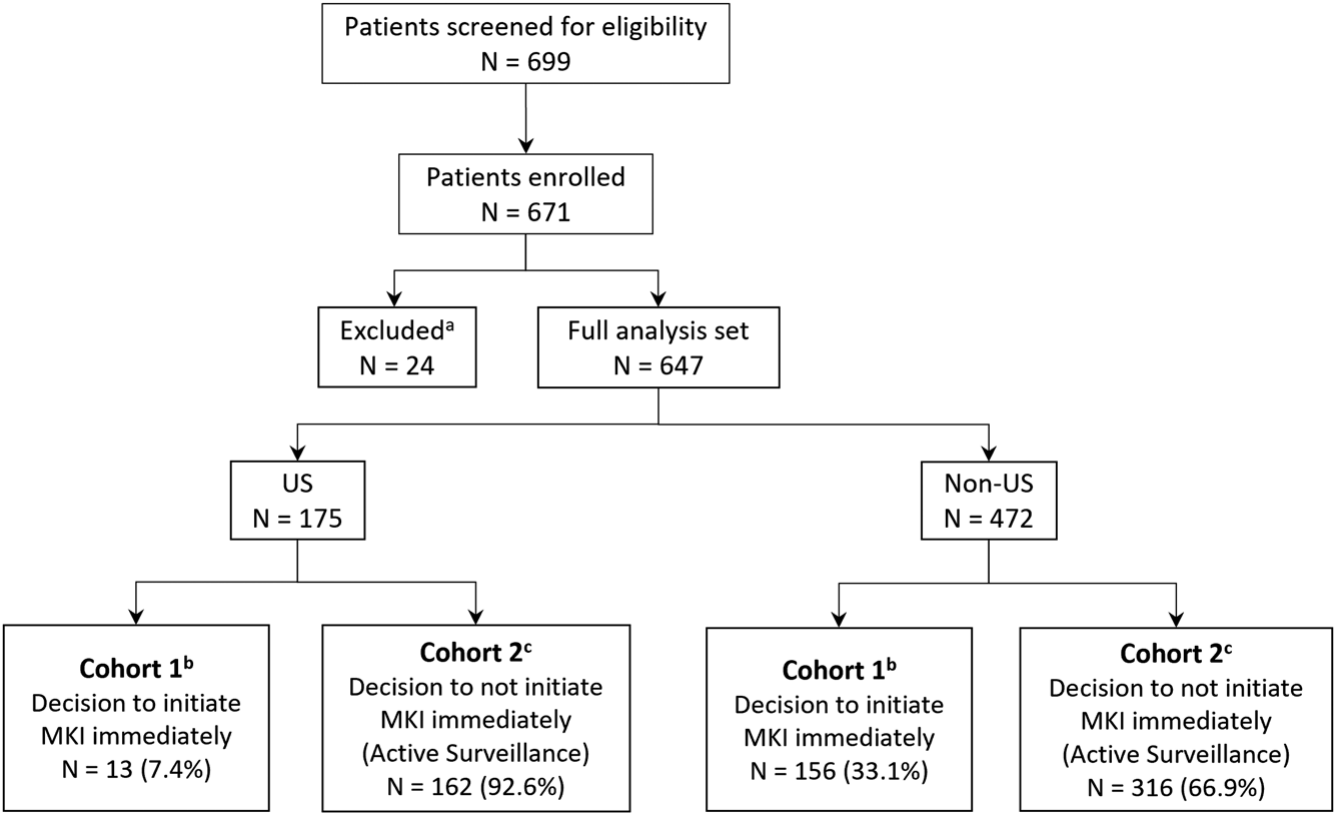
Trial profile. (A) Excluded from the full analysis set based on final data checks. 24 patients were excluded during data review because they were found to either meet exclusion criteria or did not meet the specified inclusion criteria. (B) Due to the limited enrollment of 13 patients in US cohort 1, data from this cohort could not be analyzed independently. (C) Among active surveillance patients, 41 out of 162 US patients (25%) and 124 out of 316 non-US patients (39%) later received MKI treatment.

Between April 2015 and March 2020, 671 patients from 19 countries in North America, South America, Europe, Asia, and the Middle East were enrolled in the study. Twenty-four patients were excluded for not fulfilling the criteria, leaving 647 patients evaluable for the study (175 US and 472 non-US patients) (Fig. 1). At the data cutoff (July 24, 2020), 96 (54.9%) US and 277 (58.7%) non-US patients were still being observed.

A total of 13 (7.4%) US and 156 (33.1%) non-US patients received MKI treatment at study entry (cohort 1), whereas 162 (92.6%) US and 316 (66.9%) non-US patients initially underwent active surveillance (cohort 2) (Fig. 1). Patient demographic and risk factor data are presented in Table 1.

**Table 1:** Patient demographics and risk factors. Data are presented as median (IQR) or as *n* (%).

	Overall (cohorts 1 and 2 combined)		Cohort 2 (active surveillance)	
	US	Non-US	US	Non-US
Demographics				
*n*	175	472	162	316
Age, years	68.0 (58.0–74.0)	67.0 (58.0–74.0)	68.0 (58.0–74.0)	67.0 (59.0–75.0)
≥65 years	100 (57.1)	269 (57.0)	95 (58.6)	186 (58.9)
Female	86 (49.1)	275 (58.3)	81 (50.0)	190 (60.1)
Weight, kg	84.6 (70.3–96.2)	73.0 (60.0–85.0)	83.0 (68.0–94.8)	73.0 (61.0–85.0)
ECOG PS				
0 or 1	164 (93.7)	448 (94.9)	154 (95.1)	302 (95.6)
≥2	3 (1.7)	20 (4.2)	1 (0.6)	11 (3.5)
Missing	8 (4.6)	4 (0.8)	7 (4.3)	3 (0.9)
Risk factors				
Prior thyroid disease				
Yes	47 (26.9)	72 (15.3)	44 (27.2)	57 (18.0)
No	118 (67.4)	358 (75.8)	109 (67.3)	225 (71.2)
Unknown	10 (5.7)	42 (8.9)	9 (5.6)	34 (10.8)
Radiation exposure				
Yes	12 (6.9)	17 (3.6)	12 (7.4)	12 (3.8)
No	134 (76.6)	434 (91.9)	123 (75.9)	288 (91.1)
Unknown	29 (16.6)	21 (4.4)	27 (16.7)	16 (5.1)
Inherited thyroid disorders	1 (0.6)	4 (0.8)	1 (0.6)	3 (0.9)

ECOG PS, Eastern Cooperative Oncology Group performance status.

In the US population, the median duration of observation from study entry was 32.7 months (range 0.2–57.4) in cohort 2 and 32.8 months (range 0.2–57.5) in the overall population (cohorts 1 and 2 combined). Non-US patients were observed for a similar duration.

In cohort 2, 25.3% US and 39.2% non-US active surveillance patients eventually received MKI therapy. Among them, 51.3% US and 45.9% non-US patients initiated therapy within 12 months of study entry (Table 2).

**Table 2:** Time to initiation of MKI therapy from initial visit for cohort 2 (active surveillance) patients who later received treatment. Data are presented as *n* (%).

Time to initiation	US (*n* = 41)^a,b^	Non-US (*n* = 124)^a,b^
≥1 to ≤10 days	1 (2.4)	6 (4.8)
>10 days to ≥6 months	11 (26.8)	32 (25.8)
>6 months to ≤12 months	8 (19.5)	29 (23.4)
>12 months to ≤24 months	9 (22.0)	34 (27.4)
>24 months	12 (29.3)	23 (18.5)

^a^Among active surveillance patients, 41 out of 162 US patients (25%) and 124 out of 316 non-US patients (39%) received MKI treatment; ^b^Data regarding the time to initiation of any MKI from the initial visit were unavailable for 4 US and 6 non-US active surveillance patients who eventually received MKI treatment.

MKI, multi-kinase inhibitor.

Because the sample size of US cohort 1 was insufficient for analysis, this manuscript focuses on data from the overall population and cohort 2. We compared US to non-US practices to investigate regional differences (11).

### Baseline clinical characteristics

#### US overall population

Most DTC patients presented with papillary histology and distant metastases (Table 3). In the US overall population, the median sum of the longest diameter of target lesions was 20 mm, the median number of target lesions was 2, and the median number of non-target lesions was one at study entry. According to initial ATA risk criteria, the percentage of low-to-intermediate-risk patients in the study was 37.7% (66 low-to-intermediate-risk patients/175 patients in the overall population), of which 25.1% were low-risk and 12.6% were intermediate-risk patients (Table 3). A similar percentage of high-risk (38.9%) patients were enrolled, and ATA risk status was unknown in 23.4% of patients (Table 3).

**Table 3:** Patient baseline clinical characteristics. Data are presented as median (IQR) or as *n* (%).

	**Overall**(cohorts 1 and 2 combined)		**Cohort 2**(active surveillance)	
	US (*n* = 175)	Non-US (*n* = 472)	US (*n* = 162)	Non-US (*n* = 316)
History of DTC				
Histology of DTC at initial diagnosis				
Papillary	129 (73.7)	347 (73.5)	120 (74.1)	222 (70.3)
Follicular	19 (10.9)	67 (14.2)	17 (10.5)	52 (16.5)
Hürthle cell	13 (7.4)	28 (5.9)	12 (7.4)	20 (6.3)
Poorly differentiated carcinoma	12 (6.9)	29 (6.1)	11 (6.8)	22 (7.0)
Missing	2 (1.1)	1 (0.2)	2 (1.2)	0 (0.0)
Distant metastasis at study entry				
Yes	155 (88.6)	420 (89.0)	143 (88.3)	271 (85.8)
No	13 (7.4)	43 (9.1)	13 (8.0)	37 (11.7)
Missing	7 (4.0)	9 (1.9)	6 (3.7)	8 (2.5)
ATA risk after surgery				
Low	44 (25.1)	68 (14.4)	42 (25.9)	47 (14.9)
Intermediate	22 (12.6)	145 (30.7)	21 (13.0)	90 (28.5)
High	68 (38.9)	232 (49.2)	60 (37.0)	158 (50.0)
Missing	41 (23.4)	27 (5.7)	39 (24.1)	21 (6.6)
Time from initial DTC diagnosis to study entry, months	89.1 (34.2–147.6)	80.8 (41.5–145.8)	90.2 (35.3–151.4)	80.8 (42.1–145.4)
Time from latest radiologic progression to the study entry, months	0.7 (0.2–1.9)	1.0 (0.3–2.3)	0.7 (0.2–2.0)	0.9 (0.3–2.3)
Time from RAI refractory classification to the study entry, months	17.3 (3.1–50.9)	10.5 (1.2–35.5)	19.9 (3.7–52.2)	18.4 (2.0–42.8)
Reason for classification as RAI refractory^a^				
No uptake of RAI	114 (65.1)	284 (60.2)	108 (66.7)	182 (57.6)
Progression after RAI ≤16 months	19 (10.9)	66 (14.0)	15 (9.3)	46 (14.6)
Progression after last RAI >16 months	29 (16.6)	64 (13.6)	27 (16.7)	54 (17.1)
Cumulative activity of RAI >600 mCi	8 (4.6)	28 (5.9)	8 (4.9)	20 (6.3)
Multiple reasons for RAI refractory	3 (1.7)	27 (5.7)	2 (1.2)	12 (3.8)
Missing	2 (1.1)	3 (0.6)	2 (1.2)	2 (0.6)
Tumor status at study entry				
Number of target lesions	2.0 (1.0–2.0)	2.0 (1.0–3.0)	2.0 (1.0–2.0)	2.0 (1.0–3.0)
Number of nontarget lesions	1.0 (0.0–2.5)	2.0 (0.0–5.0)	1.0 (0.0–3.0)	1.0 (0.0–4.0)
Sum of the longest diameter of target lesion(s) (mm)	20.0 (11.0–35.0)^b^	25.0 (14.9–43.0)^c^	18.0 (10.0–34.0)	24.0 (14.0–40.0)
Tumor status during study observation				
Development of new metastases during observation				
Yes	58 (33.1)	170 (36.0)	54 (33.3)	116 (36.7)
No	101 (57.7)	272 (57.6)	92 (56.8)	179 (56.6)
Missing	16 (9.1)	30 (6.4)	16 (9.9)	21 (6.6)
Disease progression during observation				
Yes	110 (62.9)	306 (64.8)	102 (63.0)	214 (67.7)
No	49 (28.0)	135 (28.6)	44 (27.2)	81 (25.6)
Missing	16 (9.1)	31 (6.6)	16 (9.9)	21 (6.6)

^a^Multiple entries possible. Percentages may not total 100 due to rounding; ^b^*n* = 127; ^c^*n* = 407

ATA, American Thyroid Association; DTC, differentiated thyroid cancer; mCi, millicurie; RAI, radioactive iodine.

Approximately 62.9% of patients progressed, and 33.1% developed new metastases during the study (Table 3). The median time from the initial DTC diagnosis to study entry was 89.1 months (IQR 34.2–147.6) (Table 3). The median time from the latest radiologic progression assessment to study entry was 0.7 months (IQR 0.2–1.9) (Table 3).

#### Comparison of US to non-US overall populations

Both US and non-US physicians enrolled a similar ratio of patients previously characterized after initial ATA risk as low-to-intermediate-risk and high-risk patients (non-US: 45.1% low-to-intermediate-risk patients; 49.2% high-risk patients); however, fewer low-risk (14.4%) and more high-risk patients (49.2%) were enrolled (Table 3) in the non-US population compared to the US population. ATA risk status was unknown in 5.7% of non-US patients. Additionally, non-US patients were enrolled in the study earlier than US patients, with a median time from the initial DTC diagnosis to study entry of 80.8 months (IQR 41.5-145.8) (Table 3). The median time from the latest radiologic progression assessment to study entry was slightly longer in the non-US (1.0, IQR 0.3–2.3) (Table 3).

#### Comparison of US and non-US cohort 2 (active surveillance) to US and non-US overall populations

No notable differences in baseline characteristics were observed between US cohort 2 and the US overall population or non-US cohort 2 vs the non-US overall population (Table 3).

### Prior RAI treatment

#### US overall population

In the US overall population, nearly all patients were treated with RAI prior to study entry (97.7%) (Table 4). Details regarding RAI treatment prior to study entry in the US population are described in Table 4.

**Table 4:** Prior and concomitant therapies. Data are presented as median (IQR) or as *n* (%).

	Overall (cohorts 1 and 2 combined)		Cohort 2^a^ (active surveillance)	
	US (*n* = 175)	Non-US (*n* = 472)	US (*n* = 162)	Non-US (*n* = 316)
Prior diagnostic or therapeutic procedure				
Yes	174 (99.4)	460 (97.5)	—	—
No	1 (0.6)	12 (2.5)		
Concomitant diagnostic or therapeutic procedure				
Yes	11 (6.3)	46 (9.7)	—	—
No	164 (93.7)	426 (90.3)		
Prior non-MKI anticancer systemic therapy				
Yes	3 (1.7)	17 (3.6)	—	—
No	172 (98.3)	455 (96.4)		
Concomitant non-MKI anticancer systemic therapy				
Yes	4 (2.3)	14 (3.0)	—	—
No	171 (97.7)	458 (97.0)		
Prior RAI treatment				
Yes	171 (97.7)	436 (92.4)	158 (97.5)	302 (95.6)
No	4 (2.3)	36 (7.6)	4 (2.5)	14 (4.4)
Prior RAI given after rhTSH				
Yes	94 (53.7)	157 (33.3)	88 (54.3)	109 (34.5)
No	77 (44.0)	279 (59.1)	70 (43.2)	193 (61.1)
Not applicable	4 (2.3)	36 (7.6)	4 (2.5)	14 (4.4)
Prior RAI given after hormone withdrawal				
Yes	50 (28.6)	300 (63.6)	45 (27.8)	209 (66.1)
No	121 (69.1)	136 (28.8)	113 (69.8)	93 (29.4)
Not applicable	4 (2.3)	36 (7.6)	4 (2.5)	14 (4.4)
Number of prior RAI treatments	2.0 (1.0–2.0)	2.0 (1.0–4.0)	1.5 (1.0–2.0)	2.0 (1.0–4.0)
Cumulative dose of prior RAI, mCi	217.0 (150.0–358.1)^b^	270.0 (150.0–450.0)^c^	213.5 (150.0–350.0)	269.2 (190.8–450.0)
Cumulative dose of RAI prior to study entry, mCi	215.1 (150.0–354.6)^b^	250.0 (150.0–430.0)^d^	212.4 (150.0–350.0)	250.0 (150.0–400.0)
Cumulative dose of prior RAI in categories				
≤600 mCi	150 (85.7)	367 (77.8)	139 (85.8)	255 (80.7)
>600 mCi	14 (8.0)	64 (13.6)	13 (8.0)	43 (13.6)
Missing	11 (6.3)	41 (8.7)	10 (6.2)	18 (5.7)
Average dose of prior RAI, mCi	151.9 (122.2–197.6)^b^	120.0 (100.0–150.0)^c^	151.6 (121.6–194.1)	123.2 (100.0–150.0)
Concomitant RAI treatment				
Yes	0 (0.0)	12 (2.5)	0 (0.0)	5 (1.6)
No	175 (100.0)	460 (97.5)	162 (100.0)	311 (98.4)

^a^Cohort 2 data are not available for (i) prior/concomitant diagnostic or therapeutic procedures and (ii) prior/concomitant non-MKI anticancer systemic therapy. ^b^*n* = 164; ^c^*n* = 431; ^d^*n* = 429.

mCi, millicurie; MKI, multi-kinase inhibitor; RAI, radioactive iodine; rhTSH, recombinant human thyrotropin.

#### Comparison of US to non-US overall populations

Several RAI practice differences were observed between the US and non-US overall populations. Slightly fewer patients received RAI prior to study entry (92.4%) in the non-US cohort (Table 4). Among patients who received RAI treatment prior to study entry, the median cumulative RAI dose received prior to study entry was higher (250 mCi, IQR 150–430), the median average RAI dose was lower (120 mCi, IQR 100–150), and the median number of RAI treatments was the same (2, IQR 1–4) (Table 4). Non-US patients demonstrated a shorter median time from RAI-R classification to study entry (10.5 months, IQR 1.2–35.5) (Table 3).

#### Comparison of US and non-US cohort 2 (active surveillance) to US and non-US overall populations

Differences in RAI administration practices were observed in US cohort 2 vs the US overall population and non-US cohort 2 vs the non-US overall population. The details of these differences are presented in Table 4.

### Efficacy

#### US overall population

**Figure 2:**
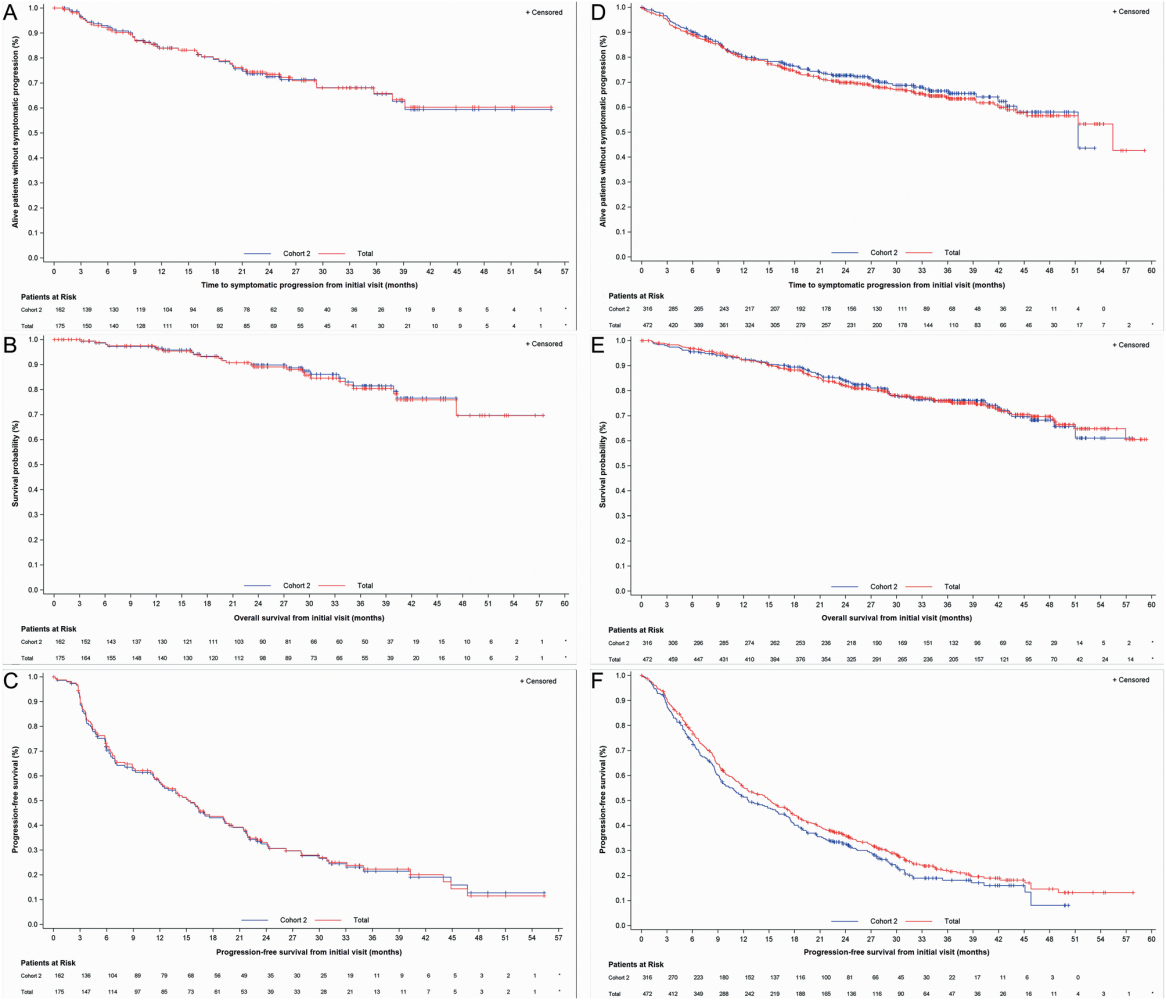
Kaplan–Meier curves for overall population for time to symptomatic progression, overall survival and progression-free survival from initial visit (US subjects *n* = 175 and non-US subjects *n* = 472). (A) US time to symptomatic progression. (B) US overall survival. (C) US progression-free survival. (D) Non-US time to symptomatic progression. (E) Non-US overall survival. (F) Non-US progression-free survival.

In the US overall population, 29 (16.6%) deaths were reported, and approximately one-quarter of the patients (*n* = 43) experienced symptomatic progression. The median TTSP and OS from study entry was not reached; therefore, the 36-month rate was assessed. The 36-month TTSP, OS, and PFS rates from study entry were 65.8% , 80.4%, and 22.3%, respectively (Table 5 and Fig. 2). Median PFS from study entry was 15.2 months (Table 5).

**Table 5:** Kaplan–Meier estimates for the overall population and cohort 2.

	Overall (cohorts 1 and 2 combined)					Cohort 2 (active surveillance)				
	*n*	Failed,^a^*n* (%)	Censored, *n* (%)	Median, months (95% CI)	36-month rate, % (95% CI)	*n*	Failed,^a^*n* (%)	Censored, *n* (%)	Median, months (95% CI)	36-month rate, % (95% CI)
Time to symptomatic progression										
US	175	43 (24.6)	132 (75.4)	NR (39.2–NE)	65.8 (55.5–74.3)	162	40 (24.7)	122 (75.3)	NR (39.2–NE)	65.6 (54.6–74.5)
Non-US	472	150 (31.8)	322 (68.2)	55.4 (45.3–NE)	63.9 (58.7–68.7)	316	92 (29.1%)	224 (70.9%)	51.4 (44.2–NE)	66.5 (60.0–72.2)
Progression-free survival										
US	175	116 (66.3)	59 (33.7)	15.2 (12.0–19.3)	22.3 (15.3–30.2)	162	108 (66.7)	54 (33.3)	15.2 (11.9–19.3)	21.5 (14.3–29.7)
Non-US	472	338 (71.6)	134 (28.4)	15.2 (12.5–17.5)	22.1 (17.9–26.5)	316	235 (74.4%)	81 (25.6%)	12.6 (9.9–16.9)	18.1 (13.4–23.4)
Overall survival										
US	175	25 (14.3)^b^	150 (85.7)	NR	80.4 (71.3–87.0)	162	22 (13.6)	140 (86.4)	NR	81.4 (71.7–88.1)
Non-US	472	116 (24.6)^b^	356 (75.4)	NR (56.9–NE)	75.8 (71.3–79.7)	316	74 (23.4%)	242 (76.6%)	NR (51.0–NE)	76.0 (70.3–80.7)

^a^The ‘failed’ number does not reflect the total number of deaths, as individuals with an unknown date of death were censored at their last known alive date.

NE, not estimable; NR, not reached.

#### Comparison of US to non-US overall populations

Similar efficacy outcomes were observed in US and non-US overall populations (Table 5 and Fig. 2); however, 128 deaths (26.9%) were reported in the non-US overall population, and one-third of the patients (*n* = 150) experienced symptomatic progression.

#### Comparison of US and non-US cohort 2 (active surveillance) to US and non-US overall populations

The 36-month TTSP, PFS, and OS rates were comparable in US cohort 2 and the US overall population (Table 5 and Fig. 2) as well as the non-US cohort 2 and the non-US overall population (Table 5 and Fig. 2).

### Cohort 2 (active surveillance) subgroup results

A subgroup analysis of cohort 2 revealed certain groups that have a poorer prognosis under active surveillance (Tables 6, 7, Supplementary Tables 1 and 2, see section on supplementary materials given at the end of this article), including patients aged ≥65 years, non-US patients with intermediate-to-high risk DTC, and US patients with low-to-intermediate-risk DTC. Regardless of the subgroup, cohort 2 patients in all regions achieved a 36-month OS rate from study entry of ≥70%.

**Table 6:** Kaplan–Meier estimates for cohort 2 (active surveillance), according to age group (years).

	US					Non-US				
	*n*	Failed, *n* (%)	Censored, *n* (%)	Median, months (95% CI)	36-month rate, % (95% CI)	*n*	Failed, *n* (%)	Censored, *n* (%)	Median, months (95% CI)	36-month rate, % (95% CI)
Time to symptomatic progression										
<65	67	19 (28.4)	48 (71.6)	39.2 (35.7–NE)	61.0 (41.8–75.6)	130	30 (23.1)	100 (76.9)	51.4 (42.8–NE)	76.6 (67.0–83.8)
≥65	95	21 (22.1)	74 (77.9)	NR	68.8 (55.4–78.9)	186	62 (33.3)	124 (66.7)	NR (33.8–NE)	59.0 (50.0–67.0)
Progression-free survival										
<65	67	46 (68.7)	21 (31.3)	12.5 (6.3–19.4)	14.0 (4.0–30.1)	130	85 (65.4)	45 (34.6)	17.6 (12.0–20.6)	26.1 (17.8–35.2)
≥65	95	62 (65.3)	33 (34.7)	16.0 (12.1–21.8)	25.3 (15.8–36.0)	186	150 (80.6)	36 (19.4)	10.3 (8.7–13.3)	13.1 (8.0–19.4)
Overall survival										
<65	67	4 (6.0)	63 (94.0)	NR	87.6 (69.1–95.3)	130	20 (15.4)	110 (84.6)	NR	85.0 (76.9–90.5)
≥65	95	18 (18.9)	77 (81.1)	NR (47.2–NE)	77.5 (64.5–86.3)	186	54 (29.0)	132 (71.0)	51.0 (48.5–NE)	69.8 (61.8–76.4)

NE, not estimable; NR, not reached.

**Table 7:** Kaplan–Meier estimates for cohort 2 (active surveillance), according to initial ATA risk.

	US					Non-US				
	*n*	Failed, *n* (%)	Censored, *n* (%)	Median, months (95% CI)	36-month rate, % (95% CI)	*n*	Failed, *n* (%)	Censored, *n* (%)	Median, months (95% CI)	36-month rate, % (95% CI)
Time to symptomatic progression										
Low	42	12 (28.6)	30 (71.4)	NR (23.7–NE)	61.0 (40.0–76.6)	47	7 (14.9)	40 (85.1)	NR (39.4–NE)	84.5 (68.0–92.9)
Intermediate	21	2 (9.5)	19 (90.5)	NR	87.8 (59.5–96.8)	90	28 (31.1)	62 (68.9)	NR (33.8–NE)	58.5 (42.5–71.5)
High	60	20 (33.3)	40 (66.7)	39.2 (21.5–NE)	59.7 (43.0–73.0)	158	49 (31.0)	109 (69.0)	51.4 (41.9–NE)	64.8 (55.5–72.7)
Missing	39	6 (15.4)	33 (84.6)	NR (35.7–NE)	74.0 (47.4–88.5)	21	8 (38.1)	13 (61.9)	42.8 (13.7–NE)	62.9 (37.2–80.5)
Progression-free survival										
Low	42	28 (66.7)	14 (33.3)	16.4 (12.9–24.1)	15.4 (4.5–32.5)	47	31 (66.0)	16 (34.0)	18.4 (8.6–31.8)	21.0 (7.6–38.7)
Intermediate	21	17 (81.0)	4 (19.0)	7.8 (4.9–17.1)	11.1 (1.9–29.8)	90	69 (76.7)	21 (23.3)	16.1 (9.2–20.5)	9.1 (3.0–19.5)
High	60	43 (71.7)	17 (28.3)	12.1 (6.7–17.0)	14.7 (5.2–28.9)	158	118 (74.7)	40 (25.3)	10.3 (8.6–13.1)	21.3 (14.8–28.6)
Missing	39	20 (51.3)	19 (48.7)	21.7 (8.9–NE)	42.2 (24.9–58.6)	21	17 (81.0)	4 (19.0)	16.9 (9.2–27.6)	13.1 (2.4–33.1)
Overall survival										
Low	42	6 (14.3)	36 (85.7)	NR (39.9–NE)	77.2 (51.9–90.3)	47	5 (10.6)	42 (89.4)	51 (NE–NE)	92.4 (78.0–97.5)
Intermediate	21	5 (23.8)	16 (76.2)	40.3 (19.4–NE)	74.2 (44.2–89.7)	90	22 (24.4)	68 (75.6)	NR	71.1 (58.8–80.4)
High	60	5 (8.3)	55 (91.7)	NR	89.1 (74.9–95.5)	158	43 (27.2)	115 (72.8)	NR (48.5–NE)	73.3 (65.0–80.0)
Missing	39	6 (15.4)	33 (84.6)	NR (47.2–NE)	80.6 (58.9–91.6)	21	4 (19.0)	17 (81.0)	NR	78.9 (52.4–91.7)

NE, not estimable; NR, not reached.

The following are the efficacy outcomes of each subgroup in the US and non-US populations.

#### Age

##### US cohort 2 (active surveillance)

In US cohort 2, patients ≥65 years old achieved a 36-month TTSP rate from study entry that was 7.8 percentage points higher and a 36-month PFS rate from study entry that was 11.3 percentage points higher than patients <65 years old; however, this did not translate to a longer OS (Table 6 and Supplementary Fig. 1). The 36-month OS rate from study entry was 10.1 percentage points lower in patients ≥65 years old (Table 6 and Supplementary Fig. 1).

##### Comparison of US to non-US cohort 2 (active surveillance)

In non-US cohort 2, patients ≥65 years old also demonstrated an OS that was 15.2 percentage points lower than patients <65 years old (Table 6 and Supplementary Fig. 2). However, in contrast to US cohort 2, the 36-month TTSP rate from study entry was 17.6 percentage points lower, and the 36-month PFS rate from study entry was 13.0 percentage points lower in patients ≥65 years old.

#### Initial ATA risk

##### US cohort 2 (active surveillance)

In US cohort 2, the intermediate-risk group achieved the highest 36-month TTSP rate from study entry compared to the low- or high-risk groups (low risk: 61.0%; intermediate risk: 87.8%; high risk: 59.7%) (Table 7 and Supplementary Fig. 3). However, the 36-month PFS rate from study entry was the lowest in the intermediate-risk group (low risk: 15.4%; intermediate risk: 11.1%; high risk: 14.7%) (Table 7 and Supplementary Fig. 3). High-risk patients achieved the highest 36-month OS rate from study entry (note, CI overlap) (Table 7 and Supplementary Fig. 3).

##### Comparison of US to non-US cohort 2 (active surveillance)

The outcomes of non-US cohort 2 patients differed from those of US cohort 2. The low-risk group demonstrated the highest 36-month TTSP rate from study entry and the highest 36-month OS rate from study entry (note, CI overlap) (Table 7 and Supplementary Fig. 4).

#### Investigator-reported sum of the target lesion diameter

##### US cohort 2 (active surveillance)

In US cohort 2, the 36-month TTSP and PFS rates from study entry were 21.3 and 10.1 percentage points lower, respectively, in patients with a higher investigator-reported sum of the target lesion diameter (≤ 24 mm vs > 24 mm) (Supplementary Table 1). However, the investigator-reported sum of the target lesion diameter did not correlate with OS (Supplementary Table 1).

##### Comparison of US to non-US cohort 2 (active surveillance)

No notable differences were observed in efficacy according to the sum of the target lesion diameter (Supplementary Table 1).

#### Prior RAI dose

Supplementary Table 2 presents the 36-month TTSP, PFS, and OS rates from study entry for US and non-US cohort 2 patients according to their previously received RAI dose (≤250 mCi vs >250 mCi).

### Efficacy of cohort 2 (active surveillance) patients with and without later MKI treatment

#### US cohort 2 (active surveillance)

**Figure 3:**
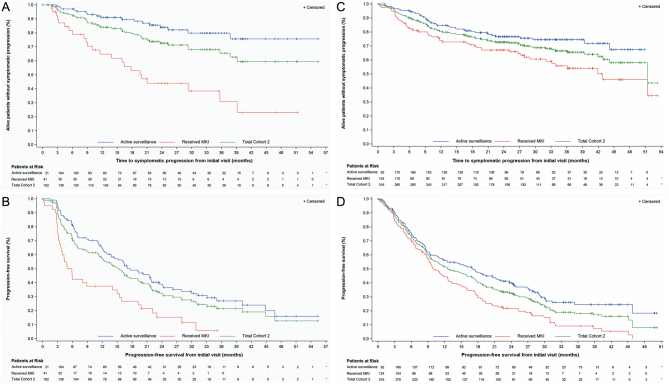
Kaplan–Meier curves for cohort 2 (active surveillance), according to MKI treatment status post enrollment for time to symptomatic progression and progression-free survival from initial visit (US subjects *n* = 162 and non-US subjects *n* = 316). (A) US time to symptomatic progression. (B) US progression-free survival. (C) Non-US time to symptomatic progression. (D) Non-US progression-free survival.

US cohort 2, patients who received MKI treatment later demonstrated a rapid decrease in PFS (Fig. 3) and a shorter median PFS (5.4 months, 95% CI: 3.7 to 14.1) compared to those who continued active surveillance (17.6 months, 95% CI: 13.8 to 21.9) (Fig. 3, Supplementary Table 3). Patients treated later also exhibited a shorter 36-month TTSP rate (30.8%, 95% CI: 13.3 to 50.2), which was a 49 percentage point decrease compared to those who continued active surveillance (79.8%, 95% CI: 68.5 to 87.5) (Fig. 3 and Supplementary Table 3).

#### Comparison of US to non-US cohort 2 (active surveillance)

Similar to US cohort 2, non-US cohort 2 patients who received MKI treatment later experienced worse outcomes than non-US patients who continued active surveillance. Median PFS was 9.9 months (95% CI: 8.7 to 12.6) in non-US patients treated later compared to 17.1 months (95% CI: 11.4 to 21.4) in non-US patients who continued active surveillance (Fig. 3 and Supplementary Table 3). The 36-month TTSP rate was 55.8% (95% CI: 45.0 to 65.3) in non-US patients treated later compared to 74.5% (95% CI: 66.7 to 80.8) in non-US patients who continued active surveillance (Fig. 3 and Supplementary Table 3).

Of interest, non-US patients treated later exhibited more favorable outcomes than their US counterparts. In the US population, patients treated later experienced faster anatomic and symptomatic progression compared to non-US-treated patients (Fig. 3 and Supplementary Table 3). This progression became evident as early as 3 months post enrollment. In contrast, non-US cohort 2 patients, irrespective of treatment status, displayed a similar progression rate for approximately 9 months post enrollment (Fig. 3). Median PFS was 5.4 months (95% CI: 3.7 to 14.1) and 9.9 months (95% CI: 8.7 to 12.6) among US and non-US patients treated later, respectively (Supplementary Table 3). The 36-month TTSP rate was 30.8% (95% CI: 13.3 to 50.2) and 55.5% (95% CI: 45.0 to 65.3) among US and non-US patients treated later, respectively (Supplementary Table 3).

## Discussion

Our study is the largest global observational study to provide real-world data regarding the treatment practices and long-term outcomes in asymptomatic patients with progressive RAI-R DTC. While the primary goal of this study was to determine when to initiate MKI treatment, we could not address this objective because of the slow accrual of events, with only 13 US patients receiving MKI treatment at study entry.

Cohort 2 (active surveillance) patients achieved a remarkable 36-month OS rate of ≥75% in the US and non-US, with 74.7% US and 60.8% non-US cohort 2 patients remaining untreated for the duration of the study. However, the cohort 2 analysis demonstrated that some patient populations may have less favorable OS with active surveillance, including patients aged ≥65 years, non-US patients with intermediate-to-high risk DTC, and US patients with low-to-intermediate-risk DTC. This finding is consistent with a prior analysis of the SELECT trial that demonstrated decreased overall survival in patients >65 years who were randomized to receive placebo with the option to cross-over to lenvatinib (12). Furthermore, we did not find investigator-reported sum of target lesion diameter (≤ 24 mm vs > 24 mm) to affect survival. It is important to note, however, that the measurement of the sum of the target lesion diameter does not accurately represent a patient’s overall tumor burden, as they omit factors such as tumor volume and lesion count. Additional data are needed to fully understand the factors that contribute to improved outcomes in certain subgroups regarding the treatment practices.

The initial sharp fall-off of PFS and TTSP in US cohort 2 patients who received MKI treatment later suggests this group may have benefited from immediate MKI treatment. Patients who were treated later experienced poorer outcomes, perhaps due to difference in disease aggressiveness that may have been apparent to the investigator at the time of enrollment. Interestingly, outcomes varied by region. In the US, patients treated later experienced more rapid progression compared to their non-US counterparts. It is possible that the shorter differences in the mean imaging interval to detect disease progression prior to enrollment observed in the US vs the non-US cohort 2 (Fig. 3 and Supplementary Table 3) may have resulted in the inclusion of US patients with disease which was growing at a faster pace. Furthermore, there may be variations in shared decision-making between patients and physicians across geographic regions, leading to differences in time to treatment initiation. Thus, additional research is needed to identify patients who are best suited for active surveillance.

Differences in practice patterns related to active surveillance were seen between the US and non-US populations. US population patients were selected for active surveillance nearly 40% more frequently, despite meeting identical criteria for MKI treatment based on radiological progression of at least one lesion ≥1 cm. Additional data, not captured in this study, are needed to gain insight into the factors influencing physicians' treatment decisions, such as the rate of disease progression at study entry (9), tumor mutational status (8), and tumor volume.

Half of US and non-US patients who qualified for MKI treatment were initially classified with ATA low-to-intermediate-risk disease. Furthermore, the US population included 50% more patients who were initial ATA low-risk compared to non-US. This underscores the importance of closely monitoring patients, regardless of their initial ATA risk status. It is important to remember that initial ATA categorization was developed to determine risk of recurrence. Grani et al. reported that the initial ATA risk accurately predicted the outcomes of DTC patients 12 months after initial treatment (13). Initial ATA risk status may be a relevant short-term predictor of prognosis; however, its predictive reliability may diminish over time.

### Limitations

This study has limitations common to observational studies, including the lack of blinding and randomization and the heterogeneity of the patient population. Selection bias is an inherent limitation of non-interventional studies. Since the decision to prescribe MKIs is subject to physician and patient preference as well as regional treatment availability the overall population and the active surveillance cohort (cohort 2) may not be comparable at baseline. Additionally, this study homogenizes regional differences, which would affect the classification of a patient’s risk status and practice patterns.

A further limitation is the insufficient collection of laboratory data to assess biochemical disease progression or adequate TSH suppression. Disease progression was assessed solely by the treating physician based on radiographic and clinical characteristics, without a centralized review board.

## Conclusions

Despite regional differences, our large global observational study suggests that active surveillance is a viable option for asymptomatic patients with progressive RAI-R DTC, as evidenced by a 36-month OS rate of ≥75%. However, for a subset of US patients, initiation of systemic therapy at the beginning of the study may have been warranted.

Patients who experienced worse outcomes under active surveillance, included US and non-US patients aged ≥65 years, non-US patients with intermediate-to-high-risk DTC, and US patients with low-to-intermediate-risk DTC. Owing to unavailable clinical information, we cannot conclude whether these populations should be treated with an MKI upfront or if regional treatment practices should be modified to achieve better outcomes.

Overall, despite regional differences, approximately 50% of US and non-US cohort 2 patients who later initiated MKI therapy did so within 12 months of study entry.

Half of US and non-US patients who qualified for MKI treatment were initially classified with ATA low-to-intermediate-risk DTC, suggesting that initial ATA classification may not accurately predict long-term outcomes.

Future research should explore factors influencing treatment decisions to distinguish between those subgroups of patients most appropriate for active surveillance from those who will benefit most from early initiation of systemic therapy.

## Supplementary materials

Supplementary Material

## Declaration of interest

The authors declare that there is no conflict of interest that could be perceived as prejudicing the impartiality of the study reported.

## Funding

This study was sponsored by Bayerhttp://dx.doi.org/10.13039/100004326 Healthcare Pharmaceuticals, Inc. The sponsor worked with the principal investigators to design the study. Data collection and interpretation, and preparation of this manuscript were performed by the investigators and the sponsor. Statistical analyses were performed by the sponsor. All authors and the sponsor approved the decision to submit the article for publication. The sponsor funded the writing.

## Author contribution statement

SB is employed by Bayer Healthcare Pharmaceuticals, Inc. AG has received consulting fees for advisory board participation from Eisai, Exelixis, and Lilly Healthcare. JC has received consulting fees for advisory board participation from Eisei, Exelixis, Merck Sharp & Dohme, and Regeneron. FW has received honorarium for participation on an advisory board with Bayer Healthcare. DB has received payment for participation on a data safety monitoring board or advisory board (support provided to institution). LW has received consulting fees from Bayer Healthcare, Eisai, Exelixis, Lilly Healthcare, Blueprint Medicines, Genentech/Roche, Merck & Co., and Pfizer. MB has received consulting fees and/or honoraria from Bayer Healthcare, Eisai, Exelixis, Ipsen, Lilly Healthcare, Blueprint Medicines; research grants and contracts from Bayer Healthcare, Lilly Healthcare, Blueprint Medicine, Eisai, and Exelixis (support provided to institution). TO has received support for attending meetings and/or travel from Daichi Sankyo and AstraZeneca; has participated on a data safety monitoring board or advisory board with Genentech/Roche, Takeda, and Novartis; has taken a leadership or fiduciary role with ASCO; and has stock or stock options in Coherus Biosciences and Gencart.
