# Supplementary material for: Real-world practice patterns and outcomes for RAI-refractory differentiated thyroid cancer
Source: Eur Thyroid J. 2024 Jan 24;13(1):e230039. doi: 10.1530/ETJ-23-0039 (PMC10831529; doi:10.1530/ETJ-23-0039)
Supplement: Supplementary Material [file supplementary_material.pdf]

Supplementary Figure 1. Kaplan-Meier curves for Cohort 2 (active surveillance), according to age group (years) for time to symptomatic progression (A), overall survival (B) and progression-free survival (C) from initial visit (US subjects N = 162)

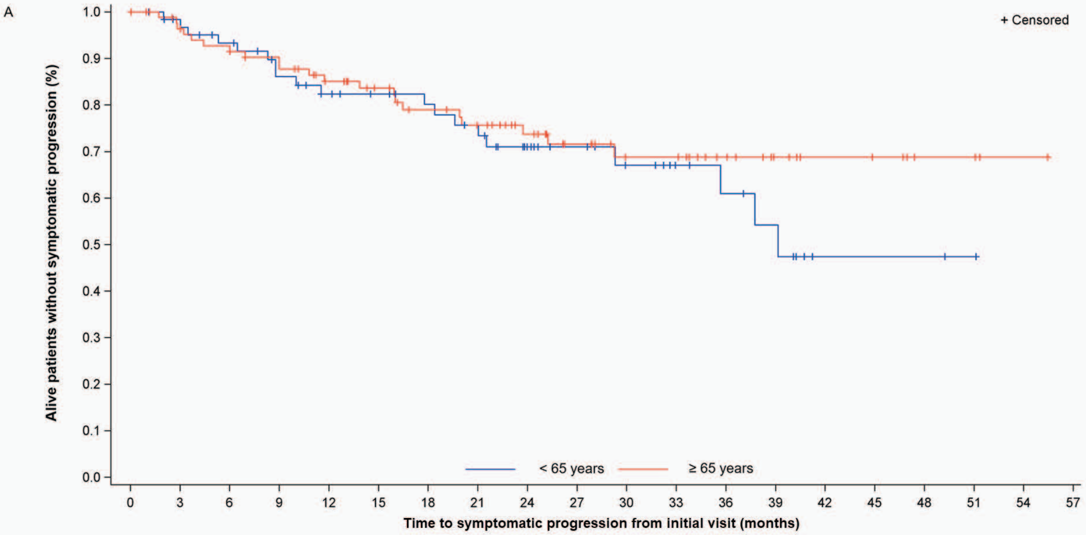

Patients at Risk

< 65 years	67	59	54	47	42	39	36	33	24	20	16	12	10	8	2	2	2	1	0	
≥ 65 years	95	80	76	72	62	55	49	45	38	30	24	24	16	11	7	6	3	3	1	*

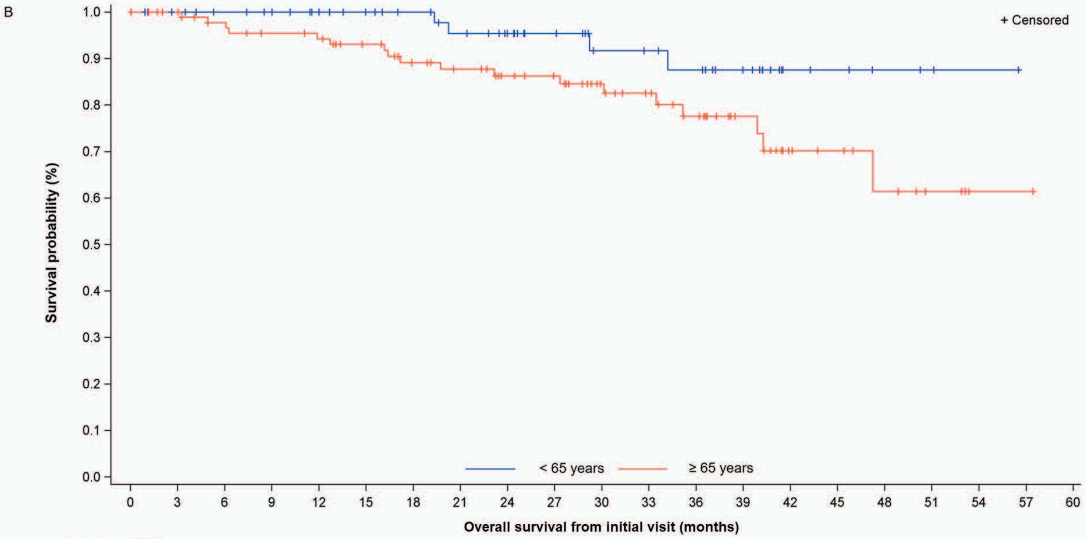

Patients at Risk

< 65 years	67	61	58	56	51	48	45	41	35	30	24	23	21	16	6	5	3	2	1	0	
≥ 65 years	95	91	85	81	79	73	66	62	55	51	42	37	29	21	13	10	7	4	1	1	*

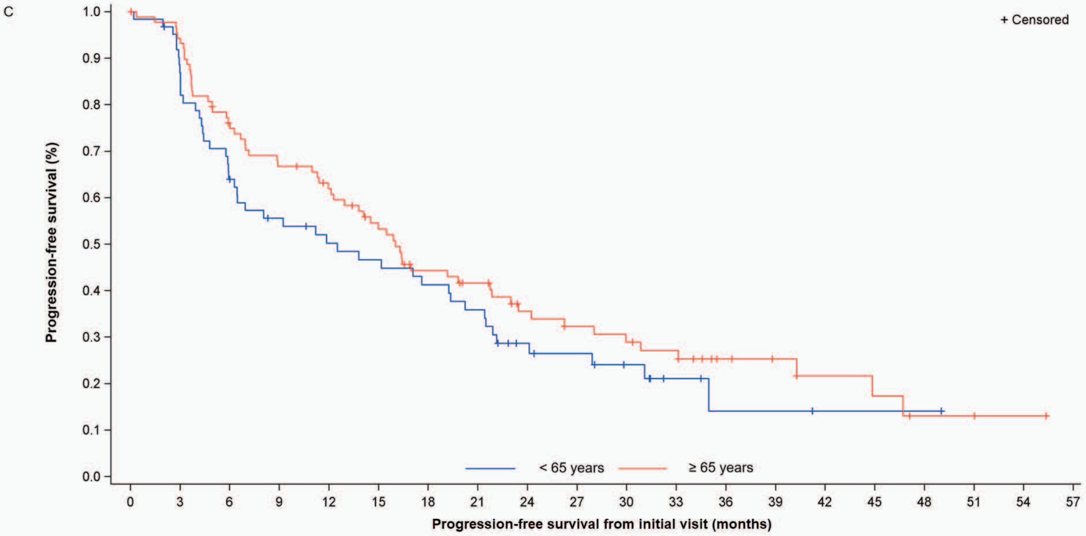

Patients at Risk

< 65 years	67	53	39	32	28	26	23	20	13	11	8	4	2	2	1	1	1	0		
≥ 65 years	95	83	65	57	51	42	33	29	22	19	17	15	9	7	5	4	2	2	1	*

Supplementary Figure 2. Kaplan-Meier curves for Cohort 2 (active surveillance), according to age group (years) for time to symptomatic progression (A), overall survival (B) and progression-free survival (C) from initial visit (Non-US subjects N = 316)

A

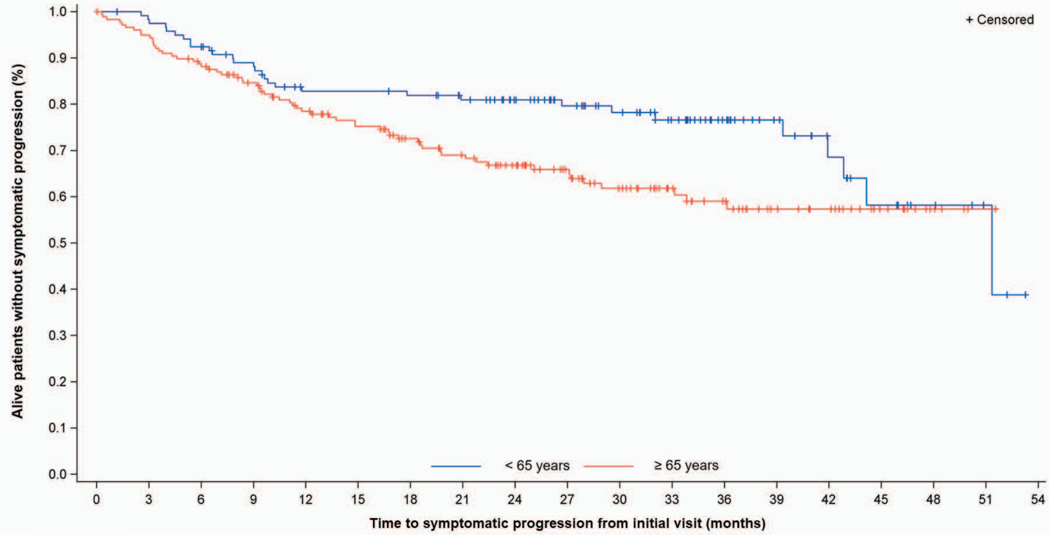

Patients at Risk

< 65 years	130	117	110	102	91	91	89	84	73	62	55	44	32	23	15	10	6	3	*
≥ 65 years	186	168	155	141	126	116	103	94	83	68	56	45	36	25	21	12	5	1	*

B

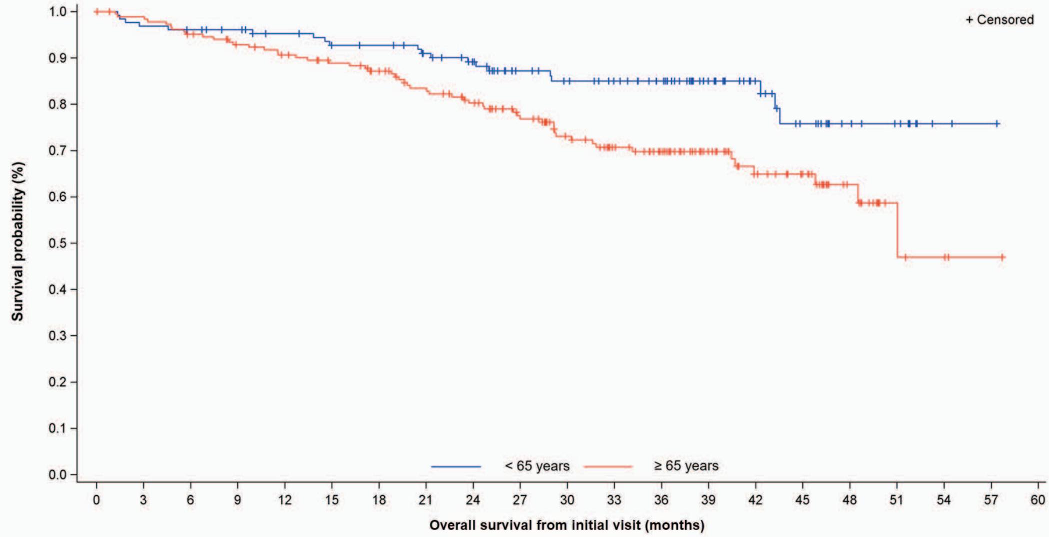

Patients at Risk

< 65 years	130	124	122	119	114	109	108	102	94	81	76	70	61	44	31	21	13	9	2	1	*
≥ 65 years	186	182	174	166	160	153	145	134	124	109	93	81	71	52	38	31	16	5	3	1	*

C

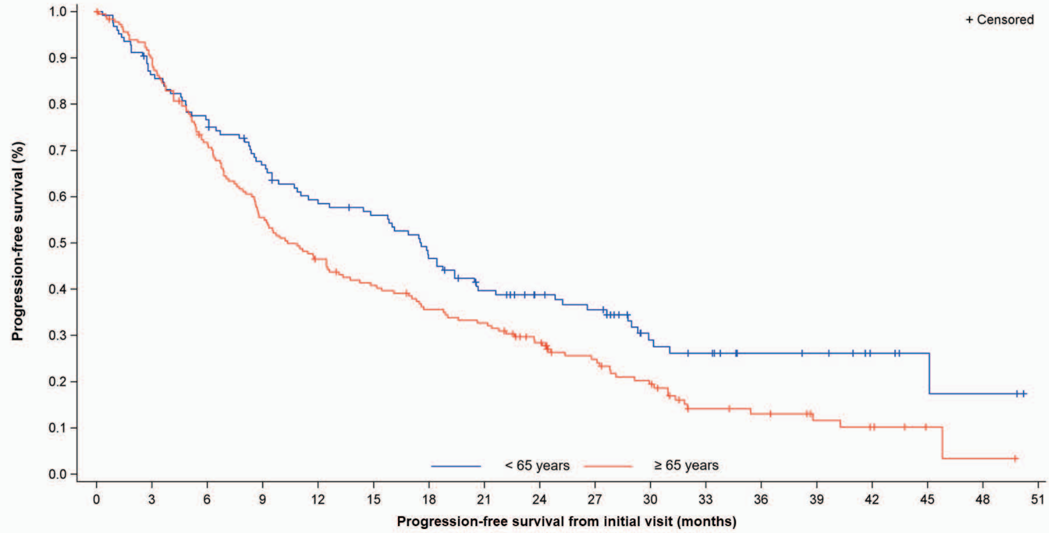

Patients at Risk

< 65 years	130	107	95	81	70	66	55	44	37	33	20	16	10	9	5	3	2	*
≥ 65 years	186	163	128	99	82	71	61	56	44	33	25	14	12	8	6	3	1	*

Supplementary Figure 3. Kaplan-Meier curves for Cohort 2 (active surveillance), according to initial ATA risk group for time to symptomatic progression (A), overall survival (B) and progression-free survival (C) from initial visit (US subjects N = 162)

A

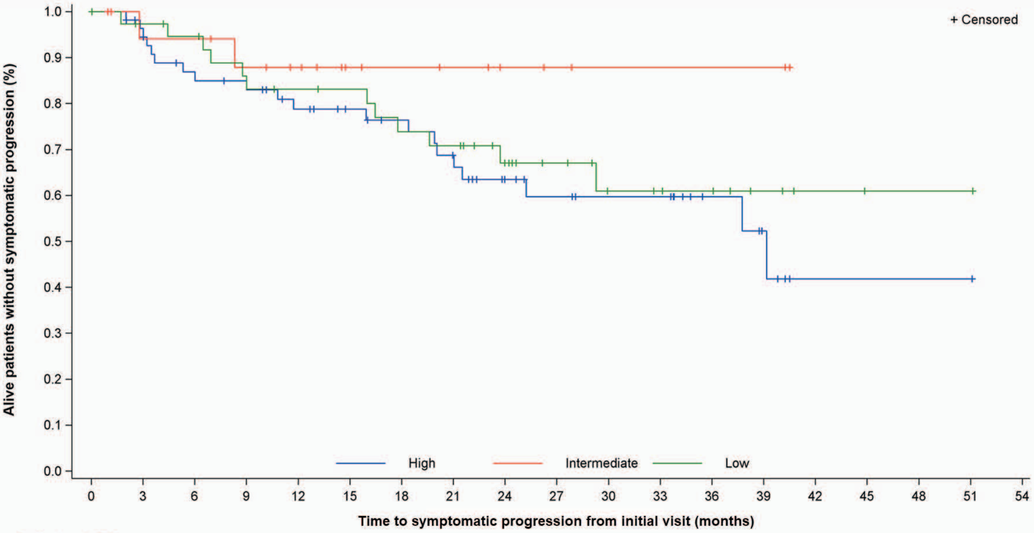

Patients at Risk																			
High	60	52	45	43	37	33	30	26	19	16	14	14	8	5	1	1	1	1	*
Intermediate	21	16	16	14	12	8	7	6	4	3	2	2	2	2	0				
Low	42	36	34	30	28	27	24	23	17	13	9	8	7	4	2	1	1	1	*

B

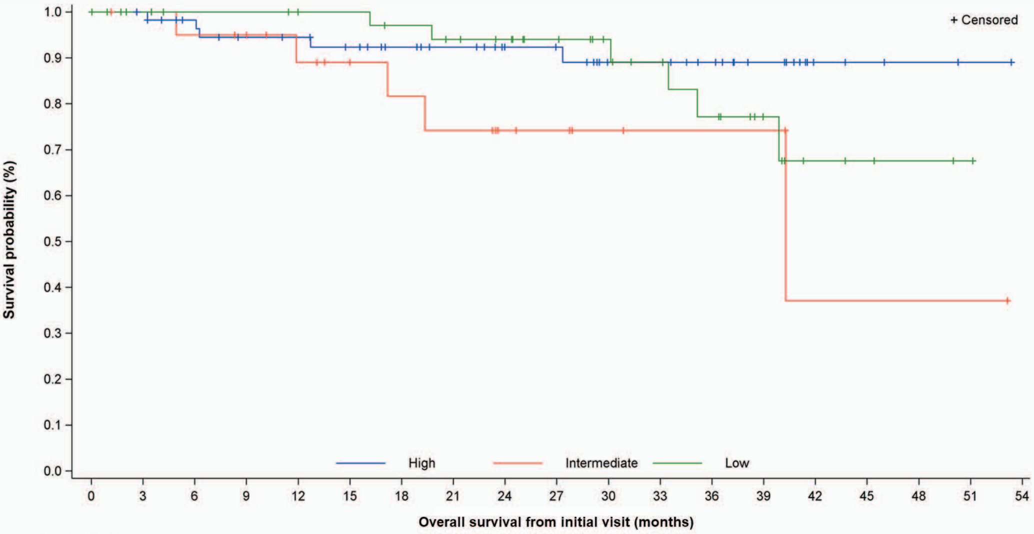

Patients at Risk																			
High	60	57	52	47	46	43	39	36	30	28	22	22	18	13	4	3	2	1	*
Intermediate	21	20	19	18	15	12	11	10	7	6	4	3	3	3	1	1	1	1	*
Low	42	38	36	36	34	34	32	30	28	23	19	16	13	8	4	3	2	1	*

C

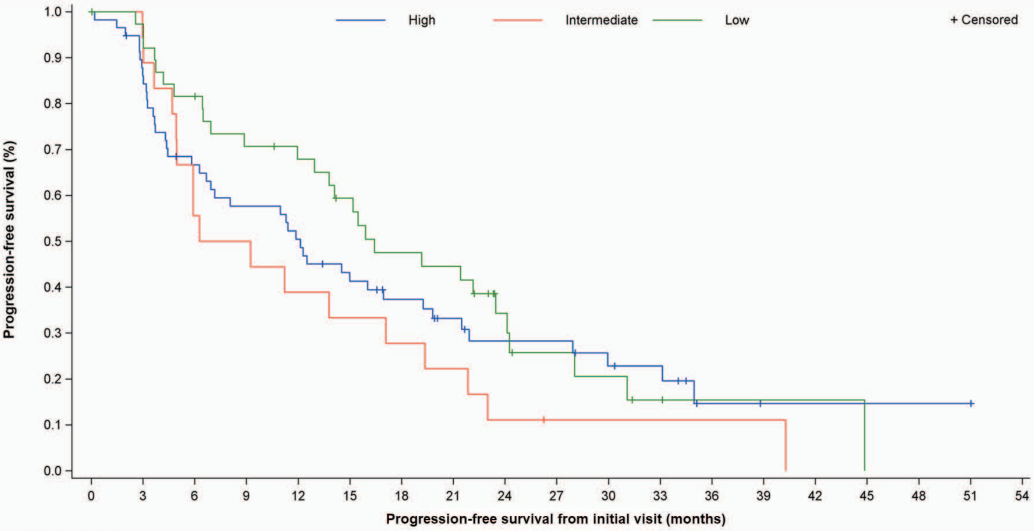

Patients at Risk																			
High	60	49	37	32	28	22	18	14	11	11	8	7	2	1	1	1	1	1	*
Intermediate	21	17	10	9	7	6	5	4	2	1	1	1	1	1	0				
Low	42	37	31	26	24	20	16	15	8	5	4	2	1	1	1	0			

Supplementary Figure 4. Kaplan-Meier curves for Cohort 2 (active surveillance), according to initial ATA risk group for time to symptomatic progression (A), overall survival (B) and progression-free survival (C) from initial visit (Non-US subjects N = 316)

A

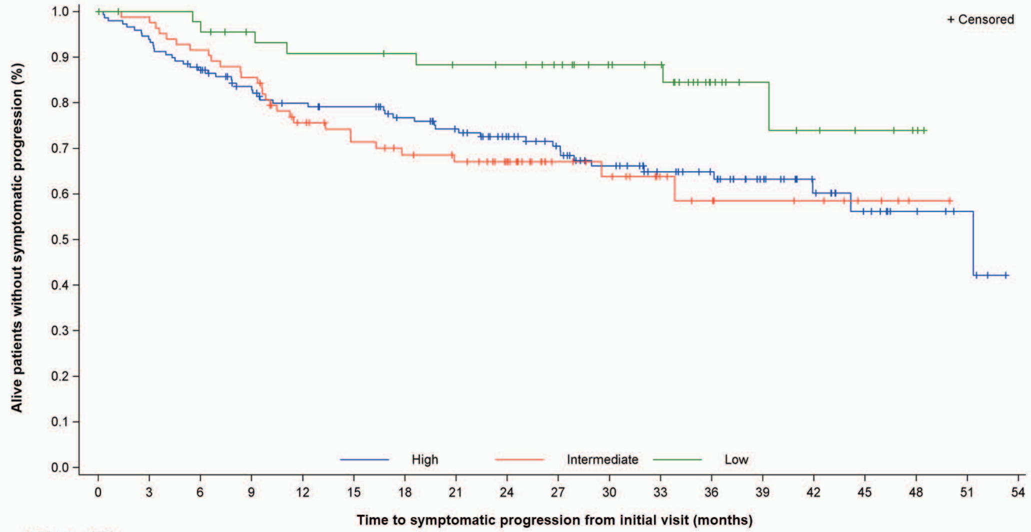

Patients at Risk

High	158	139	127	114	106	103	95	88	76	67	57	45	40	29	20	13	7	4	*
Intermediate	90	82	76	71	57	51	47	44	37	23	20	13	10	8	7	4	1	0	
Low	47	45	44	40	38	38	37	35	34	31	26	24	12	8	6	4	2	0	

B

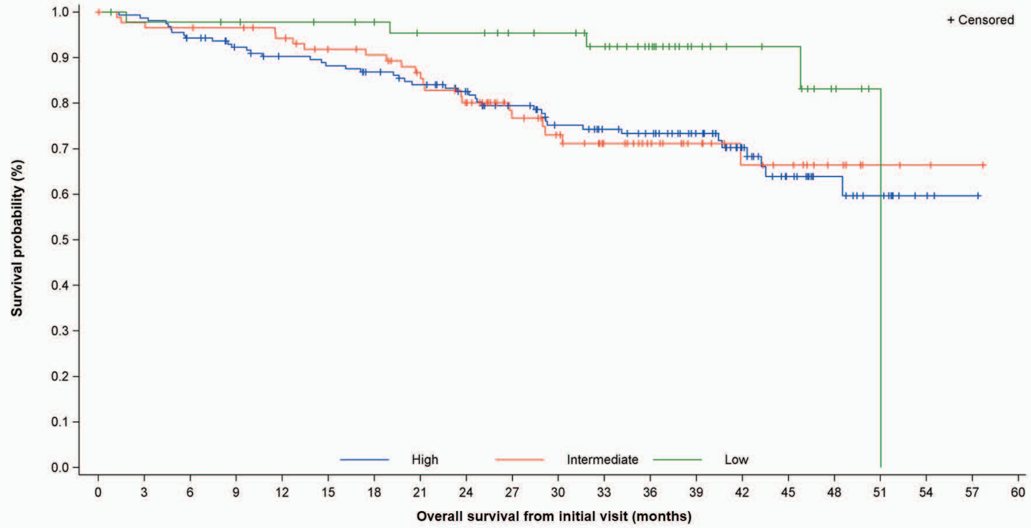

Patients at Risk

High	158	155	146	138	132	129	124	118	108	98	85	79	73	54	38	25	15	10	3	1	*
Intermediate	90	86	85	84	80	74	72	66	59	45	39	31	25	19	14	12	7	3	2	1	*
Low	47	45	45	44	43	42	41	38	38	35	34	30	23	14	11	10	5	1	0		

C

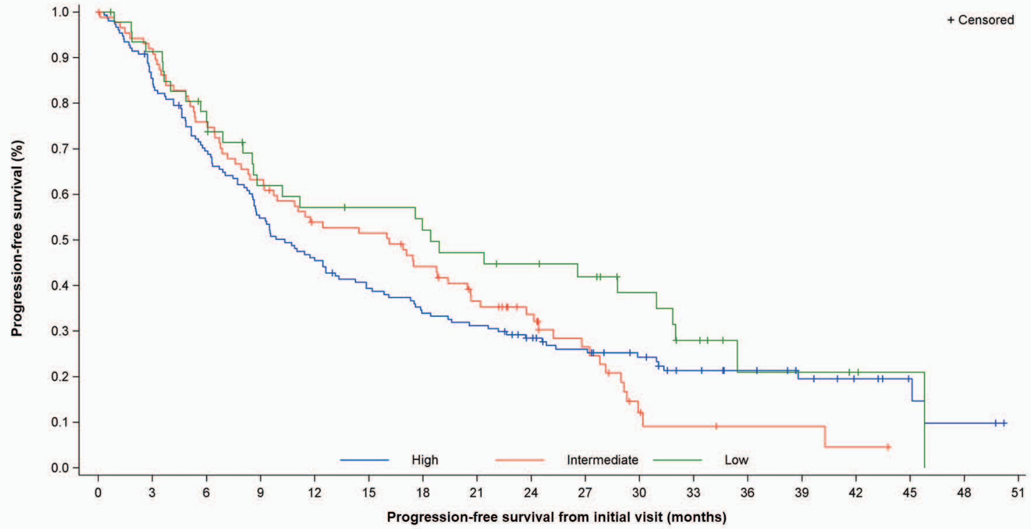

Patients at Risk

High	158	129	104	82	68	58	50	46	38	32	26	18	15	11	7	4	2	*
Intermediate	90	80	66	55	45	43	36	28	21	14	5	3	2	2	1	0		
Low	47	42	35	26	24	23	21	19	17	15	11	7	3	3	2	1	0	

Supplementary Table 1. Kaplan-Meier estimates for Cohort 2 (observation), according to target lesion size (mm) (> median versus ≤ median)

	US					Non-US				
	N	Number failed, n (%)	Number censored, n (%)	Median, months (95% CI)	36-month rate, % (95% CI)	N	Number failed, n (%)	Number censored, n (%)	Median, months (95% CI)	36-month rate, % (95% CI)
**Time to symptomatic progression**										
≤24 mm	67	14 (20.9)	53 (79.1)	NR	69.3 (51.5–81.6)	142	34 (23.9)	108 (76.1)	NR	69.5 (59.3–77.6)
>24 mm	48	19 (39.6)	29 (60.4)	25.2 (17.8–NE)	48.0 (29.1–64.6)	134	44 (32.8)	90 (67.2)	44.2 (39.4–NE)	62.8 (52.5–71.4)
Missing	47	7 (14.9)	40 (85.1)	NR	75.8 (50.7–89.3)	40	14 (35.0)	26 (65.0)	51.4 (17.8–51.4)	68.1 (49.7–81.1)
**Progression-free survival**										
≤24 mm	67	44 (65.7)	23 (34.3)	16.3 (12.3–23.0)	20.5 (9.9–33.8)	142	102 (71.8)	40 (28.2)	17.6 (12.6–21.8)	20.7 (13.4–29.1)
>24 mm	48	37 (77.1)	11 (22.9)	8.9 (5.8–16.4)	10.4 (2.5–24.7)	134	98 (73.1)	36 (26.9)	9.3 (7.7–12.5)	17.7 (10.7–26.2)
Missing	47	27 (57.4)	20 (42.6)	17.6 (7.0–33.1)	32.3 (17.7–47.7)	40	35 (87.5)	5 (12.5)	12.5 (8.3–16.9)	10.0 (2.7–23.0)
**Overall survival**										
≤24 mm	67	7 (10.4)	60 (89.6)	NR (47.2–NE)	80.8 (60.5–91.3)	142	29 (20.4)	113 (79.6)	NR	78.9 (70.4–85.2)
>24 mm	48	9 (18.8)	39 (81.3)	NR (39.9–NE)	78.2 (58.6–89.3)	134	35 (26.1)	99 (73.9)	51.0 (48.5–NE)	73.7 (64.4–80.9)
Missing	47	6 (12.8)	41 (87.2)	NR	82.7 (65.1–91.9)	40	10 (25.0)	30 (75.0)	NR (43.2–NE)	72.6 (53.4–84.9)

mm, millimeter; CI, confidence interval; NE, not estimable; NR, not reached.

Supplementary Table 2. Kaplan-Meier estimates for Cohort 2 (observation), according to cumulative RAI dose (mCi)

	US					Non-US				
	N	Number failed, n (%)	Number censored, n (%)	Median, months (95% CI)	36-month rate, % (95% CI)	N	Number failed, n (%)	Number censored, n (%)	Median, months (95% CI)	36-month rate, % (95% CI)
**Time to symptomatic progression**										
≤250 mCi	88	24 (27.3)	64 (72.7)	NR (37.7–NE)	66.2 (52.6–76.8)	151	48 (31.8)	103 (68.2)	51.4 (36.1–NE)	64.1 (54.5–72.2)
>250 mCi	64	14 (21.9)	50 (78.1)	NR (35.7–NE)	64.5 (43.4–79.4)	145	40 (27.6)	105 (72.4)	NR	68.1 (58.4–76.0)
Missing	10	2 (20.0)	8 (80.0)	NR (2.8–NE)	71.4 (25.8–92.0)	20	4 (20.0)	16 (80.0)	NR (22.4–NE)	75.2 (45.3–90.2)
**Progression-free survival**										
≤250 mCi	88	60 (68.2)	28 (31.8)	15.5 (11.4–22.1)	18.9 (9.8–30.1)	151	113 (74.8)	38 (25.2)	11.7 (9.2–15.4)	13.9 (7.4–22.3)
>250 mCi	64	42 (65.6)	22 (34.4)	13.8 (8.0–19.8)	25.1 (14.1–37.6)	145	107 (73.8)	38 (26.2)	15.8 (9.9–17.9)	20.8 (14.0–28.6)
Missing	10	6 (60.0)	4 (40.0)	16.3 (3.3–40.3)	37.0 (6.8–69.3)	20	15 (75.0)	5 (25.0)	13.4 (4.9–30.9)	25.0 (7.7–47.2)
**Overall survival**										
≤250 mCi	88	9 (10.2)	79 (89.8)	NR	82.5 (67.9–90.9)	151	39 (25.8)	112 (74.2)	NR (51.0–NE)	72.8 (64.2–79.7)
>250 mCi	64	9 (14.1)	55 (85.9)	NR	80.8 (64.4–90.1)	145	30 (20.7)	115 (79.3)	NR	79.4 (70.9–85.7)
Missing	10	4 (40.0)	6 (60.0)	40.3 (3.3–47.2)	76.2 (33.2–93.5)	20	5 (25.0)	15 (75.0)	NR (31.6–NE)	76.4 (47.6–90.7)

mCi, millicurie; CI, confidence interval; NE, not estimable; NR, not reached.

Supplementary Table 3. Kaplan-Meier estimates for Cohort 2 (active surveillance), according to MKI treatment status post-enrollment

	US					Non-US				
	N	Number failed, n (%)	Number censored, n (%)	Median, months (95% CI)	36-month rate, % (95% CI)	N	Number failed, n (%)	Number censored, n (%)	Median, months (95% CI)	36-month rate, % (95% CI)
**Time to symptomatic progression**										
Received MKI	41	23 (56.1%)	18 (43.9%)	19.9 (11.5–39.2)	30.8 (13.3–50.2)	124	50 (40.3%)	74 (59.7%)	42.8 (32.0–NE)	55.8 (45.0–65.3)
Continued surveillance	121	17 (14.0%)	104 (86.0%)	NE	79.8 (68.5–87.5)	192	42 (21.9%)	150 (78.1%)	NE	74.5 (66.7–80.8)
**Progression-free survival**										
Received MKI	41	35 (85.4%)	6 (14.6%)	5.4 (3.7–14.1)	NE	124	109 (87.9%)	15 (12.1%)	9.9 (8.7–12.6)	9.1 (4.4–16.0)
Continued surveillance	121	73 (60.3%)	48 (39.7%)	17.6 (13.8–21.9)	27.0 (17.7–37.1)	192	126 (65.6%)	66 (34.4%)	17.1 (11.4–21.4)	24.4 (17.4–32.1)
**Overall survival**										
Received MKI	41	9 (22.0%)	32 (78.0%)	47.2 (35.2–NE)	69.6 (47.3–83.9)	124	30 (24.2%)	94 (75.8%)	NE (48.5–NE)	77.4 (68.0–84.3)
Continued surveillance	121	13 (10.7%)	108 (89.3%)	NE	87.3 (77.8–92.9)	192	44 (22.9%)	148 (77.1%)	NE (51.0–NE)	75.1 (67.6–81.1)

CI, confidence interval; NE, not estimable.
